# Time to next procedure in patients with malignant pleural effusion undergoing aspiration: derivation and initial validation of the RED score

**DOI:** 10.1136/thorax-2025-223307

**Published:** 2026-01-28

**Authors:** Eleanor K Mishra, Helen Davies, Syed Hamza Abbas, Cheryl Hardy, Dominic T Beith, Dheeraj Sethi, Toshit Sapkal, Alguili Elsheikh, Asfandyar Yousuf, Emma L Hedley, Ellie Daly, Anand Sundaralingam, Dinesh Addala, Samantha A Jones, Lianne Castle, Neena Patel, Jurgen Herre, Hannah Collins, Jack Kastelik, Clare L Ross, John Corcoran, Cyrus Daneshvar, Fathimath Shiham, Alison Hufton, Geraldine A Lynch, Alex Dipper, Eleanor Barton, Amelia O Clive, Nicholas A Maskell, Allan B Clark, Najib M Rahman

**Affiliations:** 1 University of East Anglia Faculty of Medicine and Health Sciences, Norwich, UK; 2 Respiratory Medicine, Norfolk and Norwich University Hospitals NHS Foundation Trust, Norwich, UK; 3 Respiratory Medicine, Cardiff and Vale University Health Board, Cardiff, Wales, UK; 4 Norfolk and Norwich University Hospitals NHS Foundation Trust, Norwich, UK; 5 University of East Anglia, Norwich, UK; 6 Respiratory Medicine, Norfolk and Norwich University Hospital NHS Trust, Norwich, UK; 7 Oxford Centre for Respiratory Medicine, Oxford, UK; 8 Glenfield Hospital, Leicester, UK; 9 University of Oxford, Oxford, UK; 10 Respiratory Medicine, Oxford University Hospitals NHS Trust, Oxford, UK; 11 Respiratory, Oxford University Hospitals NHS Foundation Trust, Oxford, UK; 12 Cardiff and Vale University Health Board, Cardiff, UK; 13 Barts Health NHS Trust, London, UK; 14 Cambridge University Hospitals NHS Foundation Trust, Cambridge, UK; 15 Hull Royal Infirmary, Hull, UK; 16 Chest and Allergy Clinic, St Mary’s Hospital, Imperial College Healthcare NHS Trust, London, UK; 17 Interventional Pulmonology Service, Department of Respiratory Medicine, Plymouth Hospitals NHS Trust, Plymouth, UK; 18 Respiratory Medicine, Plymouth Hospitals NHS Trust, Plymouth, UK; 19 Arrowe Park Hospital, Upton, UK; 20 Southmead Hospital, Bristol, UK; 21 Academic Respiratory Unit, University of Bristol, Bristol, UK; 22 North Bristol Lung Centre, University of Bristol, Bristol, UK; 23 Norwich Medical School, University of East Anglia, Norwich, UK; 24 Oxford Respiratory Trials Unit, University of Oxford, Oxford, UK

**Keywords:** Pleural Disease, Mesothelioma, Lung Cancer

## Abstract

**Introduction:**

In patients with malignant pleural effusions (MPE), pleural fluid reaccumulates at variable rates following therapeutic aspiration. The aim of this study was to identify variables which predict time to next procedure and use them to develop a predictive score.

**Methods:**

This prospective observational cohort study in 10 British hospitals recruited patients with known or suspected malignant effusions undergoing therapeutic aspiration. Follow-up lasted 3 months and assessed time to next clinically indicated pleural procedure. Regression analysis was performed to identify independent variables predicting time to next procedure, and a score derived. Initial validation was done in two external cohorts.

**Measurements and main results:**

241 patients were recruited. Within the derivation cohort (n=180), baseline respiratory rate (R), pleural effusion depth on ultrasound (E) and dyspnoea measured using a visual analogue scale (D) (combined to form the RED score) were independent predictors of time to next procedure. Predictive models provided areas under the receiver operator curve of 0.73 and 0.75. Initial validity testing in two cohorts (n=31, n=57) demonstrated reasonable predictive value.

**Conclusions:**

In patients with MPE, baseline respiratory rate, pleural effusion depth on ultrasound and dyspnoea predict time to next procedure.

**Trial registration number:**

ISRCTN16567838.

**WHAT IS ALREADY KNOWN ON THIS TOPIC**

- Previous studies have recognised that there is variation in time to next procedure in patients with malignant pleural effusions undergoing therapeutic aspiration and have identified baseline variables (such as size of effusion) that are associated with time to next procedure but have failed to derive a predictive score.

**WHAT THIS STUDY ADDS**

- We identified that respiratory rate (R), effusion depth on ultrasound (E) and dyspnoea measured using a visual analogue scale (D) are predictive of time to next procedure. We undertook initial validation of this model in two cohorts and combined these variables to make the RED score, the first clinical score that can predict time to next procedure.

**HOW THIS STUDY MIGHT AFFECT RESEARCH, PRACTICE OR POLICY**

- The RED score uses easily available, simple clinical variables to predict time to next procedure and therefore can be used in clinical practice to predict time to next procedure. This can help clinicians and patients plan future procedures and inform their choice of procedure. Further validation work is required in other cohorts of patients.

## Introduction

 Malignant pleural effusions (MPEs) are common, with an incidence of 70 cases per 100 000 people globally per year.^1^ Breathlessness is the most common symptom and is relieved by fluid drainage.^2–4^ British Thoracic Society guidelines advise initial therapeutic aspiration (drainage of a large volume of pleural fluid), followed by a definitive procedure (chest drain and pleurodesis or indwelling pleural catheter (IPC) insertion) once the effusion reaccumulates.^5^

Time to next procedure following therapeutic aspiration for MPE varies between patients. One retrospective study found 30% of patients needed a repeat procedure by day 15, a further 18% between day 16 and 90, but 52% of patients did not have a repeat procedure within 90 days.^6^ The ability to predict when a patient might need a further procedure would help to inform patients, plan services and prevent emergency admissions, and drive practice towards earlier definitive pleural intervention in those with a high likelihood of early recurrence. Survey data from our group show that 58% of pleural physicians report patients often or sometimes are admitted as an emergency with severe breathlessness caused by recurrence, and 45% often or sometimes cancel a pleural procedure due to lack of fluid (unpublished data). 60% of patients have made at least one emergency call for urgent fluid drainage.^7^ However, both physicians and patients are unable to accurately predict time to next procedure after therapeutic aspiration.^8^

Previous retrospective studies identified variables which predict whether patients will go on to have another pleural procedure. Grosu *et al* identified predictive variables for a repeat procedure including: pleural effusion size on chest X-ray, volume of pleural fluid drained, pleural fluid lactate dehydrogenase (LDH), pleural fluid total protein (TP) and pleural fluid cholesterol.^6^ However, a predictive model failed external validation. Fysh *et al* identified that low pleural fluid pH and large effusion on chest X-ray predicted the need for a definitive procedure.^9^ Fjaellegaard *et al* found that daily pleural fluid production and large effusion size were predictive of an increased hazard of pleural fluid recurrence.^8^ Despite the identification of predictive variables, there is no validated method to predict time to next procedure. A limitation of some of these variables is that they can only be assessed once pleural fluid has been drained and therefore cannot be used to guide initial management.

We designed an observational cohort (the Reaccumulation Rate of Pleural Effusions After Therapeutic Aspiration (REPEAT) study) aimed at identifying baseline variables which predict time to next pleural procedure and deriving and doing initial validation work on a predictive score to guide management of patients with known or suspected MPE undergoing therapeutic aspiration.

## Methods

### Recruitment

This study was funded by the National Institute for Health and Care Research Research for Patient Benefit programme and prospectively registered (ISRCTN16567838). Patients with a pleural effusion who required therapeutic aspiration were recruited from 10 UK hospitals (see Online supplemental appendix for full details).^10^

### Patient characteristics

Inclusion criteria were:

- Age 18 years or over.
- Pleural effusion diagnosed on ultrasound or CT scan requiring therapeutic aspiration to relieve symptoms.
- Known or suspected malignancy as the cause of the effusion.

Both outpatients and inpatients were recruited.

Exclusion criteria were:

- Known pleural infection.
- Known transudative effusions or effusions thought to be primarily due to cardiac, renal or hepatic impairment.
- Patients requiring admission and chest drain insertion.
- Current pregnancy or breast feeding.

### Recruitment and patient flow

Patients were provided with a patient information leaflet and then gave written informed consent. There was no minimum time specified for the patient to consider trial enrolment prior to giving consent. This was to ensure that patients who were very breathless and required urgent intervention were not excluded from the study.

At baseline, all patients had clinical assessment including history and examination, routine blood tests and preprocedural radiology (including thoracic ultrasound) performed as standard care. Respiratory rate was measured as part of the baseline observations with the patient seated for enough time for this to settle to a steady rate (minimum of 2 min) and manually counted over 1 min. All thoracic ultrasounds were performed to a standard operating procedure (Online supplemental appendix). Effusion depth was measured with the patient sitting up, at the deepest point in the posterior axillary line from the parietal to the visceral pleura in centimetres.

Baseline assessment of symptoms, performance status, underlying diagnosis, current treatment and visual analogue scale for dyspnoea (VASD) and EuroQol 5-dimension 5-level (EQ-5D 5L) were recorded. The VASD is a validated measure of breathlessness in patients with pleural effusions scored from 0 (no breathlessness) to 10 (maximum possible breathlessness).^3^

### Pleural drainage

At the initial intervention, all patients underwent therapeutic aspiration until either a maximum volume (1500 mL) was drained as per British Thoracic Society guidelines, the patient developed symptoms or drainage stopped.^5^ Pleural fluid was sent for standard biochemical and other tests as clinically indicated, and a postprocedure thoracic ultrasound and chest X-ray were conducted. Patients enrolled as outpatients had the procedure performed as a day case and were only admitted if there were complications requiring admission. Inpatients were discharged when clinically appropriate.

### Follow-up

Postdischarge, all patients completed a 7-day VASD paper diary to record their level of breathlessness (online supplemental figure S1). Follow-up in person occurred at 7 days with assessment of quality of life (EQ-5D 5L), chest X-ray, thoracic ultrasound and collection of the VASD diary. Further follow-up occurred at day 30 and 3 months (either in person or by telephone) when final diagnosis, EQ-5D 5L, any further therapeutic ipsilateral pleural procedures, emergency hospital attendances, other treatment and mortality were recorded. Only patients with a final clinical diagnosis of MPE as determined by the treating clinician were included in the statistical analyses.

### Further aspiration

A further ipsilateral pleural procedure was performed if clinically indicated due to patient symptoms. Procedures which were for diagnostic purposes only (ie, image guided pleural biopsy) were not included but procedures for diagnostic and therapeutic purposes (ie, thoracoscopy) were. The type of further procedure (aspiration, drain insertion, IPC or thoracoscopy) was decided by the treating clinician. The date and type of further procedures were documented.

Participants could withdraw from the study at any time. In addition, they could be withdrawn by the investigator in the event of identification of exclusion criteria, inability to perform therapeutic aspiration or significant protocol deviation.

### Outcome measures

We initially planned to use change in size of the effusion from post drainage to day 7, measured using a validated technique, as the primary outcome measure.^11^ However, early data integrity analysis, once recruitment had been completed but prior to data lock or analysis, demonstrated that this was an unreliable measure in this cohort, with 49 missing one or both chest X-rays, 8 having 100% opacification on postprocedure chest X-ray, 2 day seven chest X-rays done following a pleural procedure and 37 showing a decrease in the size of effusion from postprocedure to day 7. These limitations were discussed with the trial management group, funder, sponsor and ethics committee and a decision was made to change the primary outcome to time to next procedure categorised as fast (next procedure in 1–13 days) versus not fast (next procedure 14 days or after or no further procedure). This outcome was chosen because it is clinically relevant and has been used in previous similar studies.^6 8 9^ Patients and clinicians agreed that this cut-off would change their management decisions. This decision was made prior to any data lock or analysis and blinded to predictor–outcome relationships. All data on time to next procedure had already been collected.

The secondary outcome was time to next procedure, categorised as slow (next procedure after 42 days/no further procedures during follow-up) versus not slow (next procedure in 1–42 days). This outcome was identified during consultation with patients and clinicians as an important time cut-off which would change their management decisions.

Therefore, patients were categorised into three groups based on time to next pleural procedure:

1. Fast: next procedure in 1–13 days.
2. Moderate: next procedure in 14–42 days.
3. Slow: next procedure after 42 days/no further procedures during follow-up.

### Sample size calculation

The study was powered on the original primary outcome measure of change in size of effusion from post drainage to day 7. A sample size of 150 participants for the derivation cohort was calculated (details in protocol). A further 30 participants were required for initial validation work. Previous feasibility data from our pilot study demonstrated that 20% of patients fulfilling these inclusion criteria did not have a final diagnosis of MPE and therefore the sample size was increased to give a total recruitment target of 240 participants. However, the change of the primary outcome measure to compare the fast and not fast groups with 10 outcomes per coefficient in the model and 78 patients in the fast group in the derivation cohort allowed us to test eight variables. Recruiting hospitals were arbitrarily divided into derivation and external validation centres based on number of patients recruited following the completion of recruitment and data lock but prior to any statistical analysis.

No patients were lost to follow-up. Patients who withdrew were excluded from analysis. Missing data were excluded from analysis.

### Predictor variables

Candidate predictor variables were chosen based on predictive variables identified in previously published studies and variables which expert pleural clinicians thought might be predictive but had not been tested in previous studies. Previous studies have identified that size of effusion is predictive of time to next procedure, which we measured on ultrasound as depth and height of effusion. We hypothesised that patients who presented as an emergency or were inpatients would have more rapid fluid production and therefore would have less time to next procedure, so these variables were assessed. Similarly, we hypothesised that patients who had had symptoms or radiological evidence of an effusion for a shorter time period had more rapid accumulation of pleural fluid and therefore would have a shorter time to next pleural procedure. Respiratory rate and breathlessness were chosen because patients who were more symptomatic might represent earlier. Finally, the neutrophil to lymphocyte ratio was chosen as a candidate predictor variable because we hypothesised that increased inflammation would lead to more rapid pleural fluid production. We chose to use variables that would be available prior to pleural fluid drainage, so did not include pleural fluid biomarkers.^6 8 9^ These variables were tested for both fast versus not fast and slow versus not slow comparisons.

### Statistical analysis

Univariate logistic regression analysis was performed for fast versus not fast and slow versus not slow groups. For the fast versus not fast comparison, patients who did not have a procedure and died prior to 14 days were excluded from the analysis. For the slow versus not slow comparison, patients who did not have a procedure and died prior to 42 days were excluded from the analysis. A fully adjusted model was estimated which included all the variables specified above. For the continuous variables, we assumed a linear association. Backwards elimination was then used to select the final covariates to be included in the final model. Residual analysis checks were undertaken to ensure that data met the key assumptions for logistic regression. These comparisons were used to create two models and two prediction equations. Hosmer-Lemeshow tests were used to assess for goodness of fit. Receiver operator curve (ROC) analysis was performed and distribution of predicted scores calculated.

The models were validated against the external validation cohort. Further validation work was performed using a second prospective ‘real world’ validation group collected as service evaluation work in the same hospitals. These patients fulfilled the same inclusion and exclusion criteria, but all data were collected as part of standard clinical care and therefore did not give individual consent. This process was approved by local information governance or audit services. The validation groups were tested against the model and skewness and kurtosis of the linear predictions determined. The intercept and slope of the ROC for the validation cohorts were derived.

A clinical score (respiratory rate (R), effusion depth on ultrasound (E) and dyspnoea measured using a visual analogue scale (D) (RED) score) was then derived from this model. The point at which patients had an equal probability of having a procedure before compared with after 14 days was set as the division between high and medium RED scores. The point at which patients had an equal probability of having a procedure at 42 days or before compared with after 42 days/no further procedure was set as the division between medium and low RED scores.

The RED score was used to analyse postprocedure outcomes in the original entire cohort, to determine whether outcomes were different for patients based on RED score. The RED score was calculated for patients with complete data for all three variables and they were then divided into high, medium and low score groups. Given that there were three groups, one way analysis of variance (ANOVA) (to compare means of normally distributed continuous variables, specifically volume of fluid drained, postprocedure effusion depth on ultrasound, pleural fluid TP, glucose, pH and LDH and improvement in breathlessness), χ² test (for categorical variables, specifically reason for stopping drainage, positive cytology for malignancy, type of cancer, benefit from drainage, time to next procedure, type of next procedure, died, procedure cancelled due to lack of fluid, emergency admission due to breathlessness caused by effusion and new oncological treatment) or Kruskal-Wallis analysis (to compare non-normally distributed continuous variables, specifically number of further procedures) were used to compare groups. Tukey’s post hoc test was used following ANOVA to determine which groups were significantly different from each other and Dunn’s test was used following Kruskal-Wallis test, with Bonferroni correction for multiple tests. Benefit from drainage was defined as a decrease in mean 7-day VASD from baseline of ≥1.4 cm based on the minimal clinically important difference.^3^

## Results

### Recruitment

Between November 2021 and June 2023, 241 patients were recruited at 10 UK hospitals (figure 1). Hospitals were arbitrarily divided into derivation and first external validation cohorts to obtain the correct number of patients in the two cohorts (180 patients in the derivation cohort and 31 in the validation cohort). Patient characteristics of the derivation and validation cohorts are summarised in table 1. Rates of missing data are summarised in the online supplement and were low; therefore, we considered that imputation was not required (online supplemental table S1).

**Figure 1:**
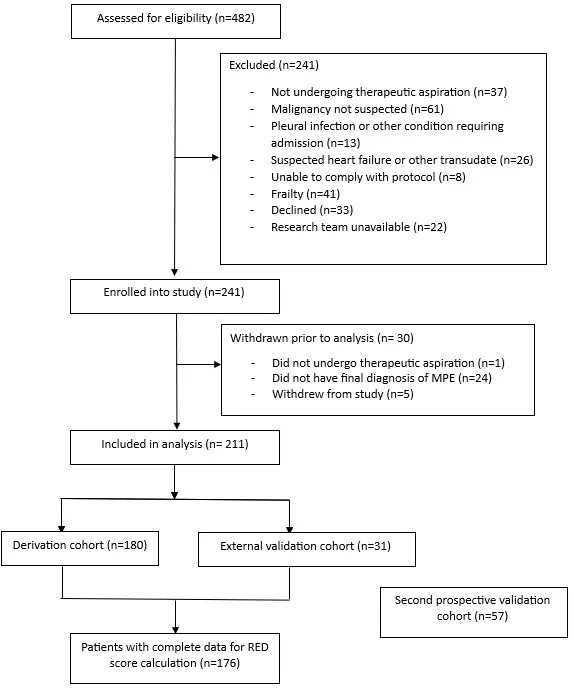
Study flow chart. RED, respiratory rate (R), effusion depth on ultrasound (E) and dyspnoea measured using a visual analogue scale (D).

**Table 1:** Characteristics of derivation, first validation and second validation cohorts

	Derivation cohort	First validation cohort	Second validation cohort
Number of participants	180	31	57
Hospital sites (number of participants recruited from that site)	Barts (9), Bristol (15), Cambridge (12) Cardiff (69), Hull (19), Imperial (11), Norfolk (27), Plymouth (18)	Oxford (8), Wirral (23)	Barts (12), Cardiff (9), Oxford (19), Wirral (8), Plymouth (9)
Age (years), mean (SD)	71.6 (12.5)	72.4 (10.1)	69.9 (13.5)
Female:male (% female)	94:86 (52.2)	13:18 (41.9)	36:21 (63.0)
Inpatient:outpatient (% inpatient)	30:150 (20.0)	5:26 (16.1)	15:42 (26.0)
Previous ipsilateral drainage procedures (yes:no) (% yes)	30:150 (20.0)	6:25 (19.4)	11:46 (19.3)
ECOG performance status (0:1:2:3:4)	25:67:56:31:1	11:15:4:1:0	5:26:11:14:1
Oxygen saturations on air (%), mean (SD)	95.5 (2.2)	95.3 (2.7)	95.4 (3.0)
Respiratory rate (breaths/min), mean (SD)	18.5 (3.4)	18.8 (2.7)	20.0 (5.3)
Preprocedure depth of effusion (cm), mean (SD)	11.6 (3.2)	12.6 (3.2)	12.6 (4.3)
Preprocedure height of effusion (cm), mean (SD)	15.5 (6.1)	18.0 (4.8)	–
Preprocedure ultrasound septation (yes:no)	10:170	4:27	–
Neutrophils:lymphocytes, mean (SD)	6.9 (6.9)	5.8 (3.4)	–
Baseline breathlessness VASD score (cm), mean (SD)	6.9 (2.3)	5.3 (3.1)	6.0 (2.9)

The second validation cohort was collected as service evaluation, therefore not all baseline variables were recorded.

cm, centimetres; ECOG, Eastern Cooperative Oncology Group; VASD, visual analogue scale for dyspnoea.

### Baseline characteristics

Characteristics of the fast versus not fast and slow versus not slow groups in the three groups are summarised in the online supplemental table S2 and S3. For the fast versus not fast comparison, there were 78 patients in the fast group and 100 in the not fast group. For the slow versus not slow comparison, there were 42 patients in the slow group and 132 patients in the not slow group.

### Recurrence groups

Variables which showed significant differences in univariate analysis between groups were respiratory rate, oxygen saturations on air, preprocedure depth and height of effusion on ultrasound and baseline breathlessness VAS score. Respiratory rate had a non-linear relationship with time to next procedure, so was categorised as <22 or ≥22 breaths/min as this cut-off was the best differentiator of time to next procedure. Performance status 3 and 4 were combined as only one individual had a value of 4. Relevant variables identified through this analysis were then investigated further in logistic regression analysis.

### Predictive derivation

For the fast versus not fast comparison, logistic regression analysis showed baseline respiratory rate, effusion depth on ultrasound and baseline VASD were independent predictors of time to next procedure (table 2).

**Table 2:** Logistic regression analysis for fast versus not fast comparison, unadjusted and fully adjusted

	Unadjusted		Fully adjusted (n=145)		Selected model (n=145)	
	OR (95% CI)	P value	OR (95% CI)	P value	OR (95% CI)	P value
Mode of presentation						
Urgent	0.74 (0.32 to 1.73)	0.49	1.09 (0.32 to 3.74)	0.90		
Routine	0.35 (0.12 to 1.05)	0.06	0.51 (0.1 to 2.77)	0.44		
Outpatient (vs inpatient)	0.55 (0.25 to 1.22)	0.14	0.52 (0.16 to 1.73)	0.29		
Duration of symptoms	1 (1 to 1)	0.32	1 (0.99 to 1)	0.25		
Time from diagnosis	1 (1 to 1)	0.58	1 (1 to 1)	0.95		
Respiratory rate group	2.53 (1.15 to 5.58)	0.021	2.47 (0.81 to 7.56)	0.11	2.8 (0.96 to 8.13)	0∙058
Preprocedure depth effusion	1.22 (1.09 to 1.36)	0.001	1.26 (1.09 to 1.44)	0.001	1.24 (1.09 to 1.41)	0.001
Neutrophil:lymphocyte	1.02 (0.98 to 1.07)	0.383	1.03 (0.95 to 1.11)	0.48		
Baseline breathlessness VAS	1.02 (1 to 1.03)	0.015	1.02 (1 to 1.04)	0.052	1.02 (1 to 1.04)	0.013
Intercept					0.01 (0.00 to 0.09)	<0.001

VAS, visual analogue scale.

This was used to create a predictive model. The fast versus not fast prediction equation was calculated as:

Log-odds=−4.52 + 1.03×(respiratory rate ≥22) + 0.22×VASD + 0.21×depth of effusion

Across the five groups, the Hosmer-Lemeshow test showed no significant difference between observed and predicted outcomes, indicating that the model provided a good fit (p=0.15). The pseudo-R^2^ in the model is 0.11. Area under the ROC was 0.73 (95% CI 0.65 to 0.82) (online supplemental figure S2).

The assumptions were checked using residual analysis and no key assumptions were invalidated.

For the slow versus not slow comparison, logistic regression analysis showed effusion depth on ultrasound and baseline VASD were independent predictors of time to next procedure (table 3).

**Table 3:** Logistic regression analysis for slow versus not slow comparison, unadjusted and fully adjusted

	Unadjusted		Fully adjusted (n=145)		Selected model (n=142)	
	OR (95% CI)	P value	OR (95% CI)	P value	OR (95% CI)	P value
Mode of presentation						
Urgent	2.07 (0.57 to 7.47)	0.27	2.78 (0.48 to 16.2)	0.26		
Routine	5.3 (1.3 to 21.5)	0.02	4.94 (0.66 to 36.9)	0.12		
Outpatient (vs inpatient)	2.11 (0.69 to 6.48)	0.19	0.84 (0.18 to 4)	0.83		
Duration of symptoms	1 (0.99 to 1)	0.339	1 (0.99 to 1)	0.47		
Time from diagnosis	1 (1 to 1)	0.968	1 (0.99 to 1)	0.41		
Respiratory rate group	0.56 (0.2 to 1.58)	0.273	0.8 (0.2 to 3.24)	0.76		
Preprocedure depth effusion	0.78 (0.68 to 0.89)	<0.001	0.77 (0.66 to 0.9)	0.001	0.77 (0.66 to 0.89)	<0.001
Neutrophil/lymphocytes,	0.97 (0.9 to 1.04)	0.366	0.97 (0.88 to 1.06)	0.48		
Baseline breathlessness VAS	0.98 (0.96 to 0.99)	0.006	0.98 (0.96 to 1)	0.036	0.79 (0.66 to 0.93)	0.006
Intercept					29.8 (3.91 to 227)	0.001

VAS, visual analogue scale.

The slow versus not slow prediction equation is:

Log-odds=3.40 − 0.24×VASD − 0.26×depth of effusion

Across the five groups, the Hosmer-Lemeshow test showed no significant difference between observed and predicted outcomes, indicating that the model provided a good fit (p=0.27). The pseudo-R^2^ in the model is 0.14. Area under the ROC was 0.75 (95% CI 0.65 to 0.84) (online supplemental figure S3).

The assumptions were checked using residual analysis and no key assumptions were invalidated.

### Validation

For the fast versus not fast comparison, the slope in the external validation cohort was 0.47 (95% CI −0.75 to 1.69) and in the second prospective validation cohort was 0.64 (95% CI 0.10 to 1.18). For the slow versus not slow comparison, the slope in the external validation cohort was 0.71 (95% CI −0.12 to 1.54) and in the second prospective validation cohort was 0.43 (95% CI 0.02 to 0.85). Further details are included in the online supplemental tables S4–S7.

These equations were combined to enable calculation of the RED score (figure 2). Of patients in the entire original cohort study, 176 had full data available to calculate the RED score. High, medium and low RED scores corresponded to being most likely to have a second procedure in 1–13 days, 14–42 days and >42 days/no further procedure, demonstrating the validity of the score (figure 3, table 4).

**Figure 2:**
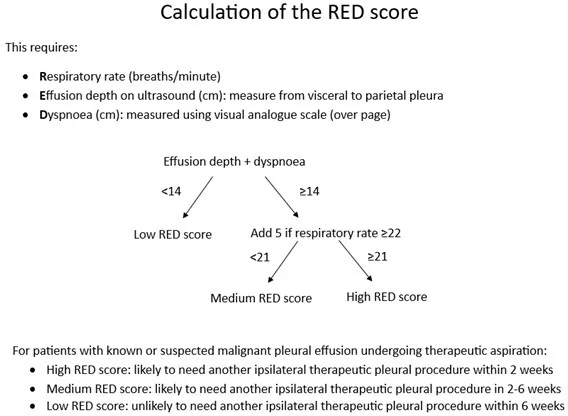
Calculation of the RED score. RED, respiratory rate (R), effusion depth on ultrasound (E) and dyspnoea measured using a visual analogue scale (D).

**Figure 3:**
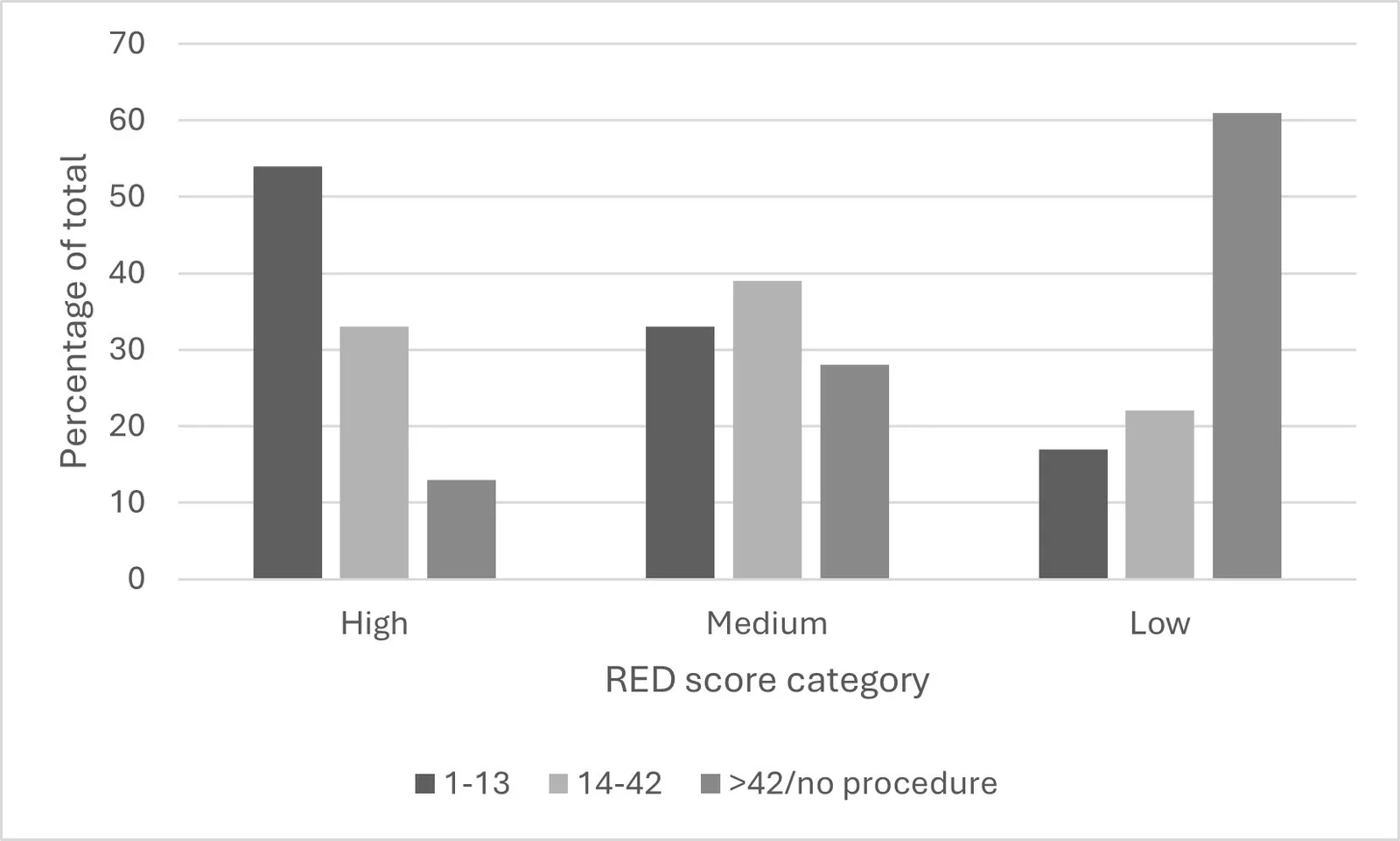
Time to next procedure by RED score in patients with complete data for RED score calculation. RED, respiratory rate (R), effusion depth on ultrasound (E) and dyspnoea measured using a visual analogue scale (D).

**Table 4:** Postprocedure outcomes of patients in the original cohort based on RED score

RED score	High	Medium	Low	P value
No.	54	104	18	
Volume fluid drained (mL), mean (SD)*	1410 (412)	1090 (471)	822 (500)	<0.001
Reason for stopping drainage†				
Target volume reached (%)	23/54 (43)	26/104 (25)	6/18 (33)	0.018
Fluid stopped draining (%)	8/54 (15)	32/104 (30)	9/18 (50)	
Patient became symptomatic (%)	21/54 (39)	44/104 (42)	2/18 (11)	
Postprocedure effusion depth on ultrasound (cm), mean (SD)*	9.4 (2.9)	5.8 (4.0)	3.9 (3.1)	<0.001
Pleural fluid total protein (g/L), mean (SD)*	44 (7.1)	42 (9.1)	42 (6.5)	0.18
Pleural fluid glucose (mmol/L), mean (SD)*	5.3 (3.0)	5.7 (2.1)	5.9 (1.9)	0.62
Pleural fluid pH, mean (SD)*	7.4 (0.13)	7.4 (0.13)	7.4 (0.079)	0.66
Pleural fluid LDH (U/L), mean (SD)*	790 (1000)	560 (770)	390 (220)	0.14
Positive cytology for malignancy (%)†	33 (61)	53 (51)	8 (44)	0.35
Type of cancer, number (%)†				
Breast	11 (20)	18 (17)	3 (17)	0.97
Lung	14 (26)	34 (33)	4 (22)	
Mesothelioma	12 (22)	17 (16)	4 (22)	
Lymphoma	2 (3.7)	6 (5.8)	1 (6.0)	
Other/unknown	15 (27)	29 (28)	6 (33)	
Improvement in breathlessness measured as decrease in VASD (cm), mean (SD)*	3.8 (2.4)	2.4 (2.2)	0.70 (3.0)	<0.001
Benefit from drainage (%)†	45/52 (87)	71/103 (69)	8/18 (44)	0.0018
Procedure 1–13 days (%)†	29 (54)	34 (33)	3 (17)	<0.001
Procedure 14–42 days (%)†	18 (33)	41 (39)	4 (22)	
Procedure >42 days or no procedure (%)†	7 (13)	29 (28)	11 (61)	
Number of further procedures, median (IQR)‡	1 (1–2)	1 (1–2)	1 (0–1)	0.005
Type of next procedure (of patients who had a further procedure)†				
Therapeutic aspiration (%)	20/50 (40)	39/81 (48)	6/10 (60)	0.75
Chest drain (%)	14/50 (28)	17/81 (21)	0/10 (0)	
Indwelling pleural catheter (%)	12/50 (24)	17/81 (21)	2/10 (20)	
Thoracoscopy (%)	4/50 (8.0)	8/81 (9.9)	2/10 (20)	
Died (%)†	14/54 (25.9)	27/104 (26.0)	4/18 (22.2)	0.71
Procedure cancelled due to lack of fluid (%)†	0/54 (0)	4/104 (4)	1/18 (6)	0.30
Emergency admission due to breathlessness caused by effusion, number (%)†	13/54 (24)	12/104 (12)	2/18 (11)	0.10
New oncological treatment, number (%)†	11/54 (20)	41/104 (39)	6/18 (33)	0.07

Benefit from drainage was defined as a decrease in mean VASD over 7 days compared with baseline of at least 1.4 cm.

* Analyses were ANOVA for continuous data.

† Χ² for categorical data.

‡ Kruskal-Wallis for number of further procedures.

ANOVA, analysis of variance; LDH, lactate dehydrogenase; RED, respiratory rate (R), effusion depth on ultrasound (E) and dyspnoea measured using a visual analogue scale (D); VAS, visual analogue scale; VASD, visual analogue scale for dyspnoea.

### Postprocedure outcomes by RED score

There was a statistically significant difference in volume of pleural fluid drained between RED score groups as determined by one-way ANOVA (*F*(2,173)=14.3, p<0.001), with patients with a higher RED score having a greater volume drained. Tukey’s post hoc test revealed that there were significant differences between the high and medium groups (mean difference 321±77 mL, p<0.001) and high and low groups (mean difference 588±124 mL, p<0.001) but no significant difference between the medium and low groups (mean difference 268±117 mL, p=0.59). Patients with a higher RED had greater effusion depth on postprocedure ultrasound (one-way ANOVA, *F*(2,83)=164, p<0.001). Tukey’s post hoc test revealed that there were significant differences between the high and medium groups (mean difference 3.5±0.84 cm, p<0.001) and high and low groups (mean difference 5.4±1.3 cm, p<0.001) but no significant difference between the medium and low groups (mean difference 1.9±1.2 cm, p=0.26). Patients with higher RED scores also had greater decreases in mean VASD following fluid drainage (one-way ANOVA, *F*(2,170)=12.3, p<0.001). Tukey’s post hoc test showed significant difference between all groups: high versus medium, mean difference 1.4±0.40 cm, p=0.002; high versus low, mean difference 3.1±0.65 cm, p<0.001; medium versus low, mean difference 1.7±0.61 cm, p=0.018. A Kruskal-Wallis H test showed that patients with a high RED score had statistically significantly more subsequent pleural procedures, χ^2^(2)=10.8, p=0.005 (table 4). Post hoc comparisons using Dunn’s test showed that this difference was significant between high and medium RED scores (adjusted p=0.041) and high and low RED scores (adjusted p=0.003) but not between medium and low RED scores (adjusted p=0.36).

When compared with combined medium and low groups, they were significantly more likely to be admitted to hospital as an emergency due to breathlessness caused by recurrence of their effusion (13/54 patients compared with 14/122, p=0.032) and less likely to start new treatment for their cancer (11/54 compared with 47/122, p=0.018). Only 44.4% of patients with a low RED score benefitted from therapeutic aspiration. There was no difference in mortality between the groups (online supplemental figure S4).

## Discussion

In this study, we have identified variables which predict time to next procedure in patients with MPE attending for therapeutic aspiration. We found that respiratory rate, effusion depth on ultrasound and breathlessness (measured by VASD) are independently predictive of time to next procedure. These variables can be easily measured in clinical practice prior to therapeutic aspiration. These have been used to develop the RED score and initial validation work has been done using both external controls and a ‘real world’ data set. The population studied has similar characteristics to other published studies on MPE and is recruited from a diverse range of hospitals. Therefore, we consider these results to be broadly applicable to patients with MPE.

We found a similar variation in time to next procedure as Grosu *et al*.^6^ As with previous studies, we found that effusion size was a predictor of time to next procedure, although we assessed this with ultrasound rather than size on chest X-ray.^8 9 12^ The other variables we identified as predictors (breathlessness and respiratory rate) were not assessed in previous studies, which may explain why Grosu was unable to validate a predictive model. The other variables identified as predictors in previous studies (volume of fluid drained, pleural fluid TP, pH, LDH and cholesterol) can only be assessed following therapeutic aspiration. They were not included in our regression analysis, because we aimed to develop a score that could be measured prior to intervention. Tumour type is often anecdotally thought to influence time to next procedure, but there was no difference in frequency of different tumour types based on RED score. Recently published data demonstrate the importance of diaphragm shape and movement in breathlessness in patients with MPE.^4 13^ The REPEAT study was conceived prior to the publication of these studies and therefore we did not assess these variables which may also influence time to next procedure.

The key limitation of this study is that we originally planned to use increase in percentage opacification comparing the postprocedure chest X-ray with the day 7 chest X-ray. The reason for initially choosing this outcome was that it was thought to be a more objective measure of pleural fluid reaccumulation than time to next procedure. It has previously been used in clinical trials of pleural effusions.^14 15^ However, this measurement was found to be unreliable to measure rate of pleural fluid reaccumulation in this cohort. This is probably because the entire hemithorax expands as a pleural effusion accumulates, with expansion of the rib cage, shift of the mediastinum and inversion of the diaphragm. Therefore, we had to change the primary outcome to time to next procedure. However, this was done prior to any other analysis. The study was sufficiently powered for the primary outcome comparing the fast and not fast groups, but was underpowered for the slow versus not slow comparison. Time to next procedure is a pragmatic outcome with clear relevance to daily clinical practice. A further limitation is that the two validation cohorts were small, meaning further validation work is required. Furthermore, the second validation cohort was collected as part of clinical practice and therefore we were unable to use cross-validation.

The areas under the curve (AUCs) of 0.75 and 0.73 indicate moderate discrimination. These values are similar to AUCs for the CURB65 score, which is used to guide antibiotic choice and hospital referral in patients with community-acquired pneumonia.^16^ The pseudo-R^2^ values are low (0.11–0.14) indicating that the RED model explains a small to moderate portion of variability in when a patient will need a repeat procedure. Other factors which affect time to next procedure include availability of staff, whether the patient is taking anticoagulant medication and need for diagnostic procedures. Currently, patients usually have a therapeutic aspiration followed by a definitive procedure once fluid reaccumulates.^5^ The ability to predict time to next procedure prior to aspiration may help clinicians and their patients make an informed choice about further management and prevent delays, for example, due to patients restarting anticoagulants or space on procedure lists. Ost *et al* showed that patients who have a second therapeutic aspiration within 14 days incur higher healthcare costs than those who have a definitive procedure; therefore, use of the RED score to plan definitive procedures could reduce costs.^17^ The RED score appears to predict important outcomes in the MPE pathway, such as emergency admissions and breathlessness relief. Research to further validate the RED score is required.

In conclusion, respiratory rate, effusion depth and predict time to next pleural procedure in patients with MPE and the RED score have been derived based on these variables. It is simple and has the potential to immediately guide patient management. Further validation is needed prior to research to determine whether use of the RED score in clinical practice improves key patient outcomes.

## Supplementary material

10.1136/thorax-2025-223307 online supplemental file 1

10.1136/thorax-2025-223307 online supplemental file 2

## Data Availability

Data are available upon reasonable request.
