# Supplementary material for: Time to next procedure in patients with malignant pleural effusion undergoing aspiration: derivation and initial validation of the RED score
Source: Thorax. 2026 Jan 28;81(10):e223307. doi: 10.1136/thorax-2025-223307 (PMC13638286; doi:10.1136/thorax-2025-223307)
Supplement: Supplementary file 1 [file thorax-81-10-s001.docx]

## Online supplement: Predicting time to next procedure in patients with malignant pleural effusion undergoing therapeutic aspiration: derivation and validation of the RED score

## Supplementary methods

## ***Standard operating procedure for thoracic ultrasound for REPEAT study***

This SOP is to be followed for pre procedure, post procedure and 1 week follow up ultrasounds:

- Sit patient on edge of couch or bed in an erect position with forearms resting on table in front of them so upper arms are in a horizontal position
- Scan using a curvilinear C2-5 probe. Hold the probe perpendicular to the chest wall
- Measurements are taken in the posterior axillary line
- Depth of effusion (see image):
  - Set depth so the deepest area of the effusion is visible
  - Scan in the posterior axillary line to find the deepest point of the effusion
  - Freeze this image and measure depth in cm from parietal pleura perpendicularly to visceral pleura.

Depth of effusion

- Height of effusion:
  - Scan in the posterior axillary line.
  - Identify the costophrenic angle (i.e the point that the diaphragm inserts into the chest wall) and mark on the skin
  - Identify the point that the visceral pleura touches the parietal pleura and mark on the skin (see images below)

- Using a disposable tape measure, measure the height between these 2 marks.
- Septated:
  - Assess subjectively whether the effusion is unseptated, mild, moderately or heavily septated (see images below).
  -

- Document the measurements on the CRF

Supplementary tables

Table S1: rates of missing data. Values represent number of missing data points for that variable for each cohort. Not all variables were collected for the second validation cohort (.. indicates that variable was not collected).

| Variable | Derivation cohort (n=180) | First validation cohort (n=31) | Second validation cohort (n=57) |
| --- | --- | --- | --- |
| Age | 0 | 0 | 0 |
| Female: male | 0 | 0 | 0 |
| Inpatient: outpatient | 0 | 0 | 0 |
| Previous ipsilateral drainage procedures | 0 | 0 | 0 |
| ECOG performance status | 0 | 0 | 0 |
| Respiratory rate | 7 | 3 | 0 |
| Pre-procedure depth of effusion | 0 | 0 | 0 |
| Pre-procedure height of effusion | 1 | 2 | .. |
| Pre-procedure ultrasound septation | 0 | 0 | .. |
| Neutrophils:lymphoctyes | 6 | 0 | .. |
| Baseline breathlessness VASD score | 22 | 5 | 0 |
| Mode of presentation | 0 | 0 | .. |
| Duration of symptoms | 3 | 0 | .. |
| Time from diagnosis | 0 | 0 | .. |
| Volume fluid drained | 0 | 0 | .. |
| Reason for stopping drainage | 1 | 0 | .. |
| Post procedure effusion depth on ultrasound | 94 | 6 | .. |
| Pleural fluid total protein | 5 | 0 | .. |
| Pleural fluid glucose | 27 | 0 | .. |
| Pleural fluid pH | 45 | 8 | .. |
| Pleural fluid LDH | 4 | 1 | .. |
| Positive cytology for malignancy | 1 | 0 | .. |
| Type of cancer | 0 | 0 | 2 |
| Post procedure breathlessness VASD (at least 1 value) | 24 | 6 | .. |
| Further procedure | 0 | 0 | 0 |
| Died | 0 | 0 | 0 |
| Emergency admission due to breathlessness | 0 | 0 | .. |
| New oncological treatment | 6 | 1 | .. |

Table S2: Descriptive statistics for the fast versus not fast analysis (i.e. patients who had their next procedure in 1-13 days versus those who had their next procedure 14 days or after/no further procedures during follow up).

|  | Derivation cohort | | External Validation cohort | | Second external validation cohort | |
| --- | --- | --- | --- | --- | --- | --- |
|  | Not fast (n=100) | Fast  (n=78) | Not fast  (n=26) | Fast  (n=5) | Not fast  (n=47) | Fast  (n=10) |
| Age (years), mean (SD) | 71∙8 (13∙3) | 71∙3 (11∙6) | 72∙3 (10∙7) | 73∙2 (7∙4) | 69.9 (14.1) | 69.8 (10.7) |
| Female: male (% female) | 53:47 (53) | 41:37 (52∙6) | 13:13 (50) | 0:5 (0) | 28 (60.0) | 8 (80) |
| Duration of symptoms prior to enrolment (days), mean (SD) | 90∙6 (180) | 69∙1 (71∙1) | 34∙7 (51∙8) | 126 (120) |  |  |
| Time from first radiological diagnosis (days), mean (SD) | 43∙5 (86∙3) | 37.0 (84∙2) | 79∙7 (189) | 52∙8 (62∙5) |  |  |
| Respiratory rate (breaths/min), mean (SD) | 17∙8 (3.0) | 19∙3 (3∙7) | 18∙8 (2∙9) | 18∙8 (1∙5) | 19.8 (5.7) | 21∙1 (3∙3) |
| Respiratory rate (breaths/min), number (%) | | | | | | |
| <22 | 85 (88) | 55 (74) | 20 (83) | 4 (100) | 37 (79) | 5 (50) |
| ≥22 | 12 (12) | 19 (26) | 4 (17) | 0 (0) | 10 (21) | 5 (50) |
| Oxygen saturations on air (%), mean (SD) | 96.0 (2∙1) | 95.0 (2∙2) | 95∙4 (2∙6) | 94∙8 (3∙8) | 95∙7 (2∙7) | 93∙8 (3∙9) |
| ECOG performance status (0:1:2:3:4) | | | | | | |
| 0 | 18 (18∙0) | 6 (7∙7) | 10 (38) | 1 (20) | 5 (11) | 0 (0) |
| 1 | 33 (33∙0) | 33 (42∙3) | 11 (42) | 4 (80) | 20 (43) | 6 (60) |
| 2 | 36 (36∙0) | 20 (25∙6) | 4 (15) | 0 (0) | 10 (21) | 1 (10) |
| 3 | 13 (13∙0) | 18 (23∙1) | 1 (4) | 0 (0) | 11 (23) | 3 (30) |
| 4 | 0 (0∙0) | 1 (1∙3) | 0 (0) | 0 (0) | 1 (2) | 0 (0) |
| Inpatient: outpatient (%) | 13:87 (13∙0) | 17:61 (21∙8) | 4:22 (15) | 1:4 (20) | 9:38 (18) | 6:4 (55) |
| Mode of presentation, number (%) | | | | | | |
| Emergency | 12 (12∙0) | 14 (17∙9) | 4 (15) | 0 (0) |  |  |
| Urgent | 66 (66∙0) | 55 (70∙5) | 13 (50) | 4 (80) |  |  |
| Routine | 22 (22∙0) | 9 (11∙5) | 9 (35) | 1 (20) |  |  |
| Pre-procedure depth of effusion (cm), mean (SD) | 10∙8 (2∙9) | 12∙6 (3∙5) | 12∙4 (3∙4) | 13∙4 (2∙4) | 12∙3 (4∙3) | 13∙6 (4∙1) |
| Neutrophils: lymphocytes, mean (SD) | 6∙5 (7∙4) | 7.0 (4∙9) | 5∙5 (3∙4) | 7∙2 (3∙3) |  |  |
| Baseline breathlessness VASD score (cm), mean (SD) | 6∙5 (2∙5) | 7∙4 (1∙9) | 5∙1 (3∙4) | 5∙7(1∙9) | 5∙5 (2∙8) | 8∙4 (2∙1) |

ECOG: Eastern Cooperative Oncology Group. SD: standard deviation. cm: centimetres. IQR: inter quartile range

Table S3: descriptive statistics for the slow versus not slow analysis (i.e. patients who had their next procedure in 1-42 days versus those who had their next procedure after 42 days or after/no further procedures during follow up).

|  | Derivation cohort | | External Validation cohort | | Second external validation cohort | |
| --- | --- | --- | --- | --- | --- | --- |
|  | Not slow (n=132) | Slow  (n=42) | Not slow  (n=18) | Slow  (n=12) | Not slow  (n=31) | Slow  (n=24) |
| Age (years), mean (SD) | 71∙6 (12∙4) | 71∙5 (13∙3) | 71∙4 (9∙5) | 73∙0 (11∙3) | 71.9 (11∙9) | 67∙4 (15∙0) |
| Sex, number female (%) | 68 (51∙5%) | 24 (57∙1%) | 6 (33%) | 7 (58%) | 17 (61%) | 16 (67%) |
| Duration of symptoms prior to enrolment (days), mean (SD) | 87∙5 (157) | 63∙3 (93∙4) | 54∙8 (85∙5) | 42∙8 (56∙2) |  |  |
| Time from first radiological diagnosis (days), mean (SD) | 41∙3 (91∙1) | 41∙9 (68∙8) | 115 (222) | 20∙7 (28∙0) |  |  |
| Respiratory rate (breaths/min), mean (SD) | 18∙5 (3∙7) | 18∙1 (2∙5) | 19∙2 (3∙3) | 18∙1 (1∙7) | 20∙6 (5∙6) | 19∙0 (5∙4) |
| Respiratory rate (breaths/min), number (%) | | | | | | |
| <22 | 101 (80∙2) | 36 (87∙8) | 13 (81.3) | 10 (90.9) | 19 (68) | 21 (88) |
| ≥22 | 25 (19∙8) | 5 (12∙2) | 3 (18.6) | 1 (9.1) | 9 (32) | 3 (12) |
| Oxygen saturations on air (%), mean (SD) | 95∙5 (2∙2) | 95∙6 (2∙2) | 94∙7 (3∙0) | 96∙4 (2∙1) | 94∙6 (3∙2) | 96∙5 (2∙2) |
| ECOG performance status, number (%) | | | | | | |
| 0 | 16 (12∙1) | 8 (19∙0) | 4 (22) | 7 (58) | 2 (7) | 3 (12) |
| 1 | 52 (39∙4) | 13 (31∙0) | 10 (56) | 5 (42) | 13 (46) | 12 (50) |
| 2 | 39 (29∙5) | 15 (35∙7) | 4 (22) | 0 (0) | 7 (25) | 4 (17) |
| 3 | 24 (18∙2) | 6 (14∙3) | 0 (0) | 0 (0) | 6 (21) | 5 (21) |
| 4 | 1 (0∙8) | 0 (0) | 0 (0) | 0 (0) | 0 (0) | 0 (0) |
| Inpatient: outpatient (% inpatient) | 24:108 (18∙2) | 4:38 (9∙5) | 2:16 (11) | 2:10 (17) | 10:21 (36) | 2:22 (8) |
| Mode of presentation, number (%) | | | | | | |
| Emergency | 22 (16∙7) | 3 (7∙1) | 1 (6) | 2 (17) |  |  |
| Urgent | 92 (69∙7) | 26 (61∙9) | 10 (56) | 7 (58) |  |  |
| Routine | 18 (13∙6) | 13 (31∙0) | 7 (39) | 3 (25) |  |  |
| Pre-procedure depth of effusion (cm), mean (SD) | 12∙1 (3∙2) | 10∙0 (3∙2) | 12∙8 (3∙3) | 12∙0 (3∙2) | 13∙7 (4∙2) | 12∙0 (4∙2) |
| Neutrophils: lymphocytes, mean (SD) | 6∙9 (7∙0) | 5∙8 (4∙5) | 5∙9 (3∙1) | 5∙5 (4∙0) |  |  |
| Baseline breathlessness VASD score (cm), mean (SD) | 7∙2 (21∙4) | 6∙0 (2∙6) | 6∙1 (2∙5) | 3∙3 (3∙9) | 6∙9 (2∙9) | 5∙2 (2∙6) |

ECOG: Eastern Cooperative Oncology Group. SD: standard deviation. cm: centimetres.

Table S4: Descriptive statistics of the linear predictions (log-odds) for the fast versus not fast comparison.

|  | Mean | Standard deviation | Skewness | Kurtosis |
| --- | --- | --- | --- | --- |
| Derivation cohort | -0∙48 | 0∙90 | 0∙016 | 2∙75 |
| First validation cohort | -0∙48 | 1∙1 | -0∙64 | 2∙4 |
| Second validation cohort | -0∙27 | 1∙5 | 0∙48 | 2∙3 |

Table S5: Validation of fast versus not fast model.

|  | Area under LROC curve | Intercept | Slope |
| --- | --- | --- | --- |
| Derivation cohort | 0∙73 | X | X |
| First validation cohort | 0∙55 | -1∙5 | 0∙47 |
| Second validation cohort | 0∙75 | -1.6 | 0∙64 |

LROC: localization receiver operating characteristic

Table S6: Descriptive statistics of the linear predictions (log-odds) for the slow versus not slow comparison:

|  | Mean | Standard deviation | Skewness | Kurtosis |
| --- | --- | --- | --- | --- |
| Derivation cohort | -1∙2 | 0∙98 | 0∙29 | 2∙9 |
| First validation cohort | -1∙4 | 1∙8 | 0∙30 | 2∙2 |
| Second validation cohort | -1∙5 | 1∙5 | -0∙44 | 2∙5 |

Table S7: Validation of slow versus not slow model.

|  | Area under LROC curve | Intercept | Slope |
| --- | --- | --- | --- |
| Derivation cohort | 0∙75 | X | X |
| First validation cohort | 0∙72 | -0∙45 | 0∙71 |
| Second validation cohort | 0∙69 | 0∙46 | 0∙43 |

LROC: localization receiver operating characteristic

Supplementary figures

Figure S1: REPEAT study VASD diary. VAS lines were 10cm when printed.

Figure S2: receiver operating characteristic (ROC) analysis comparing fast versus not fast groups in derivation cohort

Figure S3: receiver operating characteristic (ROC) analysis for slow versus not slow groups in derivation cohort

Figure S4: time to death by RED score
